# Around the Hospitals

**Published:** 1892-05-28

**Authors:** 


### AROUND THE HOSPITALS.

**The Children's Hospital, Paddington Green.**—The annual meeting of this institution was held on the 25th inst. The card calling the meeting had a street plan on the back showing the nearest underground stations, and contained an urgent invitation to Governors to attend. This is an excellent idea and should be generally adopted with the object of increasing the attendance at these meetings. The report takes a new departure by giving a complete plan of the ground floor of the present building, and the proposed extensions. It would be an immense service to the Governors, the hospitals, and all concerned, if similar plans were to be given in the annual report by hospitals generally. The institution is doing a most useful work and deserves much more extended support than it obtains at present. We are glad to see that the congregation of Christ Church, Lancaster Gate, is warmly interested in its welfare, and we hope that the Rev. C. J. Ridgeway may be able, as we know him to be willing, to arouse his congregation sufficiently to promptly secure the £5,000 which the Committee are urgently in want of at the present time.

**Evelina Hospital for Sick Children.**—The annual meeting of this institution was recently held, and the wards of the hospital were thrown open for inspection. As soon as the necessary funds are provided, it is proposed to enlarge the building by adding a wing. The number of beds has recently been increased to sixty-six, but in view of the demands made upon them, the Committee are anxious to provide one hundred beds. It is proposed also to open additional wards in the new wing, with dormitories for the nurses, and to establish a nursing institution in connection with the hospital. During the year 727 children have been treated as in-patients and 5,463 as out-patients. Of these 114 were sent to convalescent homes at the cost of the convalescent fund. The hospital was established in 1869 by Baron F. de Rothschild, to treat sick children of the poor without distinction of creed. The cots are nearly always fully occupied, and from fifty to a hundred out-patients attend daily. Unfortunately, the trustees have had to sell out stock to enable the work of the hospital to be carried on during the year. An urgent appeal is therefore made for additional funds.

**Liverpool Royal Infirmary.**—At present, the buildings, with the exception of the sleeping accommodation for the staff (which is surprisingly bad as we pointed out in a descriptive article last year), and also the administration are as good as those of any institution in the world. Liverpool may well be proud of its Infirmary and of its most capable resident staff. In addition to the ordinary wards of a general hospital this institution has provided pay wards, ophthalmic wards, and a lock hospital. Its chapel is probably the most beautiful building of the kind in the country and attracts a number of strangers from outside to the Sunday services. We are sorry to see that the Committee have not met with the support from the public which they require, and we would suggest that they should make arrangements to throw open the hospital to public inspection for two days in three consecutive weeks and to invite the people of Liverpool to come and see how excellent are the arrangements. Such a step would be calculated to awaken public interest, and should lead to a large increase in the annual subscription list. Having regard to the importance of the nursing department of this institution we shall hope to see in future years that the report contains a special report by the Matron, Miss Stains, who is one of the oldest and ablest Lady Superintendents in the country. The private wards produced an income of £294 in 1891, and this sum will no doubt be largely increased from year to year.

